# 2-[(*E*)-2-(4-Chloro­phen­yl)ethen­yl]-1-methyl­pyridinium 4-bromo­benzene­sulfonate

**DOI:** 10.1107/S1600536809026968

**Published:** 2009-07-18

**Authors:** Suchada Chantrapromma, Kullapa Chanawanno, Hoong-Kun Fun

**Affiliations:** a Crystal Materials Research Unit, Department of Chemistry, Faculty of Science, Prince of Songkla University, Hat-Yai, Songkhla 90112, Thailand; b X-ray Crystallography Unit, School of Physics, Universiti Sains Malaysia, 11800 USM, Penang, Malaysia

## Abstract

In the title compound, C_14_H_13_ClN^+^·C_6_H_4_BrO_3_S^−^, the cation exists in an *E* configuration with respect to the ethenyl bond and is almost planar, the dihedral angle between the pyridinium and the benzene rings being 2.80 (7)°. The dihedral angles between the benzene ring of the anion and the pyridinium and benzene rings of the cation are 80.88 (7) and 79.05 (7)°, respectively. In the crystal, the cations are stacked into columns along the *a* axis as a result of π–π inter­actions between the pyridinium and chloro­benzene rings with a *Cg*⋯*Cg* distance of 3.6976 (8) Å. The anions are linked into chains along the *a* axis by weak C—H⋯O inter­actions. These anion chains are linked to adjacent cations by additional weak C—H⋯O and C—H⋯Br inter­actions, forming a two-dimensional network parallel to the *ab* plane. There are also short O⋯Br [3.2567 (11) Å] and C⋯O [2.9917 (18) Å] contacts. The crystal structure is further stabilized by C—H⋯π inter­actions involving the aromatic ring of the anion.

## Related literature

For bond-length data, see: Allen *et al.* (1987 ▶). For background to non-linear optical materials research, see: Andreu *et al.* (1999 ▶); Jagannathan *et al.* (2007 ▶); Cho *et al.* (2002 ▶); Lakshmanaperumal *et al.* (2003 ▶); Veiros (2001 ▶). For related structures, see: Chanawanno *et al.* (2008 ▶; 2009 ▶); Chantrapromma *et al.* (2006 ▶); Fun *et al.* (2009 ▶). For the stability of the temperature controller used in the data collection, see: Cosier & Glazer (1986 ▶).
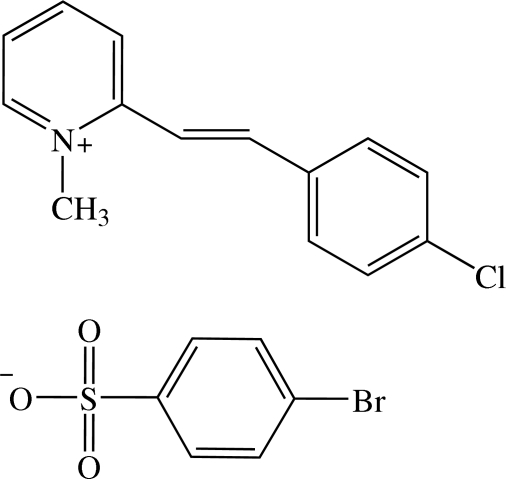


## Experimental

### 

#### Crystal data

C_14_H_13_ClN^+^·C_6_H_4_BrO_3_S^−^
*M* *_r_* = 466.77
Monoclinic, 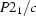

*a* = 7.9476 (1) Å
*b* = 18.6099 (3) Å
*c* = 12.7173 (2) Å
β = 93.467 (1)°
*V* = 1877.50 (5) Å^3^
*Z* = 4
Mo *K*α radiation
μ = 2.46 mm^−1^
*T* = 100 K
0.51 × 0.26 × 0.24 mm

#### Data collection

Bruker APEXII CCD area-detector diffractometer
Absorption correction: multi-scan (*SADABS*; Bruker, 2005 ▶) *T*_min_ = 0.368, *T*_max_ = 0.586 (expected range = 0.348–0.554)
36425 measured reflections
8227 independent reflections
6744 reflections with *I* > 2σ(*I*)
*R*_int_ = 0.026

#### Refinement

*R*[*F* ^2^ > 2σ(*F* ^2^)] = 0.031
*wR*(*F* ^2^) = 0.079
*S* = 1.02
8227 reflections
245 parameters
H-atom parameters constrained
Δρ_max_ = 1.30 e Å^−3^
Δρ_min_ = −0.73 e Å^−3^

### 

Data collection: *APEX2* (Bruker, 2005 ▶); cell refinement: *SAINT* (Bruker, 2005 ▶); data reduction: *SAINT*; program(s) used to solve structure: *SHELXTL* (Sheldrick, 2008 ▶); program(s) used to refine structure: *SHELXTL*; molecular graphics: *SHELXTL*; software used to prepare material for publication: *SHELXTL* and *PLATON* (Spek, 2009 ▶).

## Supplementary Material

Crystal structure: contains datablocks global, I. DOI: 10.1107/S1600536809026968/sj2633sup1.cif

Structure factors: contains datablocks I. DOI: 10.1107/S1600536809026968/sj2633Isup2.hkl

Additional supplementary materials:  crystallographic information; 3D view; checkCIF report

## Figures and Tables

**Table 1:** Hydrogen-bond geometry (Å, °)

*D*—H⋯*A*	*D*—H	H⋯*A*	*D*⋯*A*	*D*—H⋯*A*
C1—H1*A*⋯Br1^i^	0.93	2.89	3.6585 (14)	141
C3—H3*A*⋯O3^ii^	0.93	2.53	3.4285 (17)	163
C6—H6*A*⋯O2^iii^	0.93	2.55	3.4702 (17)	172
C7—H7*A*⋯O1^iv^	0.93	2.51	3.4057 (17)	161
C13—H13*A*⋯O2^iii^	0.93	2.49	3.4205 (17)	180
C14—H14*A*⋯O1	0.96	2.49	3.3383 (17)	148
C14—H14*C*⋯O2^iii^	0.96	2.48	2.9917 (18)	113
C17—H17*A*⋯O1^v^	0.93	2.28	3.2110 (17)	175
C20—H20*A*⋯O1	0.93	2.55	2.9157 (17)	104
C10—H10*A*⋯*Cg*3^vi^	0.93	2.77	3.6831 (16)	169
C12—H12*A*⋯*Cg*3^iii^	0.93	2.79	3.6402 (16)	153

Symmetry codes: (i) 
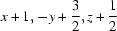
; (ii) 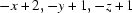; (iii) 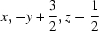; (iv) 
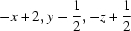
; (v) 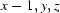; (vi) 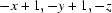. *Cg*3 is the centroid of the C15–C20 ring.

**Fig. 1.**
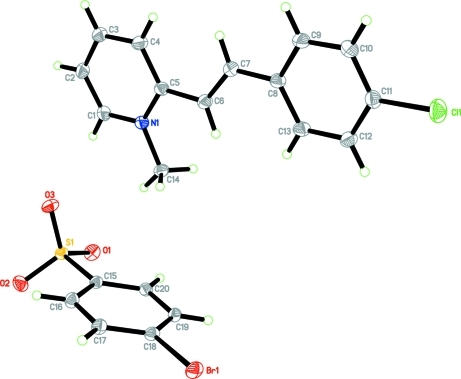
The molecular structure of the title compound, with 50% probability displacement ellipsoids and the atom-numbering scheme.

**Fig. 2.**
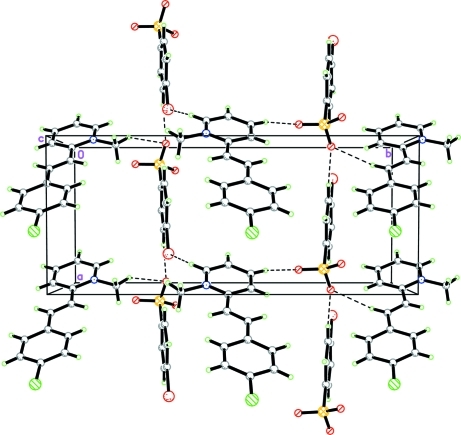
The crystal packing of the title compound viewed down the c axis. Weak C—H···O and C—H···Br interactions are shown as dashed lines.

## supplementary crystallographic information

### Comment

Stilbazolium is a competitive candidate for organic nonlinear optical (NLO) materials and yields high optical nonlinearities including large second harmonic generation (SHG) (Andreu *et al.*, 1999; Jagannathan *et al.*, 2007). Molecules with large π systems have been extensively used in attempts to obtain NLO materials (Veiros, 2001). This hypothesis led to a popular approach towards such materials which are synthesized from compounds with extended π conjugated systems to ensure large second-molecular hyperpolarizability (*β*) (Lakshmanaperumal *et al.*, 2003). However, this approach will not be effective if the molecules of these compounds are arranged in centrosymmetric space groups (Cho *et al.*, 2002). The title compound (I) has been synthesized and its crystal structure was undertaken in order to establish the orientation of molecules in the solid state. It was found that (I) crystallized in centrosymmetric space group *P*2_1_/*c* so no second-order nonlinear optical properties are observed.

In the molecule of the title compound, C_14_H_13_ClN^+^. C_6_H_4_BrO_3_S^-^ (Fig. 1), the cation exists in an *E* configuration with respect to the ethenyl C6═C7 bond [1.3393 (19) Å] and the torsion angle of C5–C6–C7–C8 = 178.74 (14)°. The cation is almost planar with the dihedral angle between the pyridinium and benzene rings of the cation being 2.80 (7)°. The anion is inclined to the cation which is reflected by the dihedral angles between the benzene ring of the anion and the pyridinium and benzene rings of the cation being 80.88 (7) and 79.05 (7)°. The Br substituents are coplanar with the attached benzene rings. The bond distances in both cation and anion have normal values (Allen *et al.*, 1987) and are comparable with the closely related compounds (Chanawanno *et al.*, 2008; 2009; Chantrapromma *et al.*, 2006; Fun *et al.*, 2009).

In the crystal packing (Fig. 2), all O atoms of the sulfonate group are involved in weak C—H···O interactions (Table 1). The cations are stacked into columns along the *a* axis as a result of π–π interactions with a Cg_1_···Cg_2_ distance of 3.6976 (8) Å (symmetry code 2-x, 1-y, -z); Cg_1_ and Cg_2_ are the centroids of the C1–C5/N1 and C8–C13 rings, respectively. The anions are linked into chains along the *a* axis by a weak C—H···O interaction (Table 1). The anions are linked to the adjacent cations by weak C—H···O and C—H···Br interactions forming a 2D network parallel to the *ab* plane. There are also O···Br [3.2567 (11) Å; symmetry codes: 1+x, 3/2-y, 1/2+z and -1+x, 3/2-y, -1/2+z] and C···O [2.9917 (18) Å; symmetry code: x, 3/2-y, z] short contacts. The crystal structure is further stabilized by C—H···π interactions involving the C15–C20 ring of the anion. .

### Experimental

2-[(*E*)-2-(4-Chlorophenyl)ethenyl]-1-methylpyridinium iodide (0.24 g, 0.67 mmol) which was prepared according to a previous report (Chanawanno *et al.*, 2008) was mixed (1:1 molar ratio) with silver(I) 4-bromobenzenesulfonate (0.23 g, 0.67 mmol) (Chantrapromma *et al.*, 2006) in methanol solution (100 ml). The mixture solution was stirred for 30 min, the precipitate of silver iodide which formed was filtered and the filtrate was evaporated to give the title compound as an orange solid. Brown block-shaped single crystals of the title compound suitable for *x*-ray structure determination were recrystallized from methanol by slow evaporation at room temperature over a month, Mp. 508-509 K.

### Refinement

All H atoms were positioned geometrically and allowed to ride on their parent atoms, with d(C-H) = 0.93 Å for aromatic and CH and 0.96 Å for CH_3_ atoms. The *U*_iso_ values were constrained to be 1.5*U*_eq_ of the carrier atom for methyl H atoms and 1.2*U*_eq_ for the remaining H atoms. A rotating group model was used for the methyl groups. The highest residual electron density peak is located at 0.70 Å from Cl1 and the deepest hole is located at 0.50 Å from Cl1.

### Crystal data

**Table:**

C_14_H_13_ClN^+^·C_6_H_4_BrO_3_S^−^	*F*(000) = 944
*M**_r_* = 466.77	*D*_x_ = 1.651 Mg m^−^^3^
Monoclinic, *P*2_1_/*c*	Melting point = 508–509 K
Hall symbol: -P 2ybc	Mo *K*α radiation, λ = 0.71073 Å
*a* = 7.9476 (1) Å	Cell parameters from 8227 reflections
*b* = 18.6099 (3) Å	θ = 2.2–35.0°
*c* = 12.7173 (2) Å	µ = 2.46 mm^−^^1^
β = 93.467 (1)°	*T* = 100 K
*V* = 1877.50 (5) Å^3^	Block, brown
*Z* = 4	0.51 × 0.26 × 0.24 mm

### Data collection

**Table:**

Bruker APEXII CCD area-detector diffractometer	8227 independent reflections
Radiation source: sealed tube	6744 reflections with *I* > 2σ(*I*)
graphite	*R*_int_ = 0.026
φ and ω scans	θ_max_ = 35.0°, θ_min_ = 2.2°
Absorption correction: multi-scan (*SADABS*; Bruker, 2005)	*h* = −12→12
*T*_min_ = 0.368, *T*_max_ = 0.586	*k* = −29→30
36425 measured reflections	*l* = −20→20

### Refinement

**Table:**

Refinement on *F*^2^	Primary atom site location: structure-invariant direct methods
Least-squares matrix: full	Secondary atom site location: difference Fourier map
*R*[*F*^2^ > 2σ(*F*^2^)] = 0.031	Hydrogen site location: inferred from neighbouring sites
*wR*(*F*^2^) = 0.079	H-atom parameters constrained
*S* = 1.02	*w* = 1/[σ^2^(*F*_o_^2^) + (0.0355*P*)^2^ + 1.227*P*] where *P* = (*F*_o_^2^ + 2*F*_c_^2^)/3
8227 reflections	(Δ/σ)_max_ = 0.002
245 parameters	Δρ_max_ = 1.30 e Å^−^^3^
0 restraints	Δρ_min_ = −0.73 e Å^−^^3^

### Special details

**Table:**

Experimental. The crystal was placed in the cold stream of an Oxford Cryosystems Cobra open-flow nitrogen cryostat (Cosier & Glazer, 1986) operating at 100.0 (1) K.
Geometry. All esds (except the esd in the dihedral angle between two l.s. planes) are estimated using the full covariance matrix. The cell esds are taken into account individually in the estimation of esds in distances, angles and torsion angles; correlations between esds in cell parameters are only used when they are defined by crystal symmetry. An approximate (isotropic) treatment of cell esds is used for estimating esds involving l.s. planes.
Refinement. Refinement of F^2^ against ALL reflections. The weighted R-factor wR and goodness of fit S are based on F^2^, conventional R-factors R are based on F, with F set to zero for negative F^2^. The threshold expression of F^2^ > 2sigma(F^2^) is used only for calculating R-factors(gt) etc. and is not relevant to the choice of reflections for refinement. R-factors based on F^2^ are statistically about twice as large as those based on F, and R- factors based on ALL data will be even larger.

### Fractional atomic coordinates and isotropic or equivalent isotropic displacement parameters (Å^2^)

**Table:**

	*x*	*y*	*z*	*U*_iso_*/*U*_eq_	
Br1	0.233790 (18)	0.807999 (8)	0.089929 (11)	0.01901 (4)	
Cl1	0.39854 (5)	0.44980 (2)	−0.33199 (3)	0.02829 (8)	
S1	0.87153 (4)	0.769209 (17)	0.42302 (2)	0.01344 (6)	
N1	1.03961 (15)	0.57489 (6)	0.26973 (9)	0.0163 (2)	
O1	1.01963 (13)	0.79162 (6)	0.36935 (9)	0.01957 (19)	
O2	0.83179 (15)	0.81603 (6)	0.50948 (8)	0.0220 (2)	
O3	0.87526 (14)	0.69338 (5)	0.45166 (8)	0.01917 (19)	
C1	1.1310 (2)	0.57574 (8)	0.36310 (11)	0.0206 (3)	
H1A	1.1703	0.6194	0.3904	0.025*	
C2	1.1670 (2)	0.51371 (8)	0.41839 (11)	0.0219 (3)	
H2A	1.2302	0.5150	0.4823	0.026*	
C3	1.1070 (2)	0.44893 (8)	0.37682 (11)	0.0214 (3)	
H3A	1.1279	0.4063	0.4134	0.026*	
C4	1.01612 (19)	0.44842 (8)	0.28073 (11)	0.0195 (2)	
H4A	0.9780	0.4050	0.2521	0.023*	
C5	0.98054 (17)	0.51241 (7)	0.22572 (11)	0.0163 (2)	
C6	0.88146 (18)	0.51613 (7)	0.12614 (11)	0.0180 (2)	
H6A	0.8578	0.5612	0.0973	0.022*	
C7	0.82227 (19)	0.45815 (7)	0.07362 (11)	0.0191 (2)	
H7A	0.8488	0.4134	0.1027	0.023*	
C8	0.71911 (18)	0.45944 (7)	−0.02595 (11)	0.0166 (2)	
C9	0.6540 (2)	0.39406 (7)	−0.06589 (11)	0.0207 (3)	
H9A	0.6790	0.3518	−0.0293	0.025*	
C10	0.5528 (2)	0.39117 (8)	−0.15891 (12)	0.0225 (3)	
H10A	0.5082	0.3477	−0.1836	0.027*	
C11	0.51988 (19)	0.45417 (8)	−0.21391 (11)	0.0193 (2)	
C12	0.58160 (19)	0.52002 (8)	−0.17711 (11)	0.0193 (2)	
H12A	0.5572	0.5619	−0.2149	0.023*	
C13	0.68025 (18)	0.52247 (7)	−0.08315 (11)	0.0184 (2)	
H13A	0.7211	0.5664	−0.0578	0.022*	
C14	1.0017 (2)	0.64493 (7)	0.21800 (11)	0.0202 (3)	
H14A	1.0547	0.6828	0.2591	0.030*	
H14B	0.8820	0.6524	0.2126	0.030*	
H14C	1.0439	0.6449	0.1488	0.030*	
C15	0.69849 (16)	0.77983 (7)	0.32849 (10)	0.0132 (2)	
C16	0.53518 (17)	0.77816 (7)	0.36296 (10)	0.0166 (2)	
H16A	0.5188	0.7707	0.4339	0.020*	
C17	0.39682 (17)	0.78748 (7)	0.29222 (11)	0.0162 (2)	
H17A	0.2879	0.7859	0.3150	0.019*	
C18	0.42424 (17)	0.79926 (7)	0.18665 (10)	0.0146 (2)	
C19	0.58535 (18)	0.80181 (7)	0.15040 (10)	0.0150 (2)	
H19A	0.6012	0.8102	0.0796	0.018*	
C20	0.72348 (17)	0.79154 (7)	0.22238 (10)	0.0139 (2)	
H20A	0.8323	0.7925	0.1993	0.017*	

### Atomic displacement parameters (Å^2^)

**Table:**

	*U*^11^	*U*^22^	*U*^33^	*U*^12^	*U*^13^	*U*^23^
Br1	0.01657 (7)	0.02094 (6)	0.01858 (6)	0.00174 (5)	−0.00664 (5)	0.00000 (5)
Cl1	0.02706 (18)	0.0357 (2)	0.02110 (15)	0.00237 (15)	−0.00652 (13)	−0.00412 (14)
S1	0.01243 (13)	0.01367 (12)	0.01385 (12)	0.00116 (10)	−0.00235 (10)	−0.00005 (9)
N1	0.0170 (5)	0.0155 (5)	0.0163 (5)	0.0024 (4)	0.0002 (4)	−0.0001 (4)
O1	0.0124 (4)	0.0235 (5)	0.0225 (5)	−0.0018 (4)	−0.0013 (4)	0.0022 (4)
O2	0.0244 (5)	0.0241 (5)	0.0169 (4)	0.0055 (4)	−0.0041 (4)	−0.0065 (4)
O3	0.0203 (5)	0.0150 (4)	0.0215 (4)	0.0013 (4)	−0.0043 (4)	0.0040 (3)
C1	0.0247 (7)	0.0205 (6)	0.0162 (5)	0.0026 (5)	−0.0010 (5)	−0.0015 (4)
C2	0.0251 (7)	0.0248 (6)	0.0154 (5)	0.0050 (5)	−0.0009 (5)	0.0015 (5)
C3	0.0249 (7)	0.0205 (6)	0.0190 (6)	0.0057 (5)	0.0023 (5)	0.0034 (5)
C4	0.0209 (6)	0.0164 (5)	0.0212 (6)	0.0017 (5)	0.0007 (5)	0.0013 (4)
C5	0.0153 (6)	0.0161 (5)	0.0173 (5)	0.0026 (4)	−0.0003 (4)	0.0000 (4)
C6	0.0196 (6)	0.0169 (5)	0.0171 (5)	0.0012 (5)	−0.0020 (5)	0.0007 (4)
C7	0.0226 (6)	0.0170 (6)	0.0176 (5)	0.0008 (5)	0.0003 (5)	0.0013 (4)
C8	0.0169 (6)	0.0146 (5)	0.0183 (5)	−0.0003 (4)	−0.0001 (4)	−0.0001 (4)
C9	0.0261 (7)	0.0141 (5)	0.0217 (6)	−0.0008 (5)	−0.0021 (5)	−0.0002 (4)
C10	0.0278 (7)	0.0174 (6)	0.0220 (6)	−0.0026 (5)	−0.0016 (5)	−0.0034 (5)
C11	0.0186 (6)	0.0221 (6)	0.0170 (5)	0.0015 (5)	−0.0004 (5)	−0.0023 (4)
C12	0.0197 (6)	0.0176 (6)	0.0203 (6)	0.0012 (5)	−0.0014 (5)	0.0018 (4)
C13	0.0182 (6)	0.0144 (5)	0.0223 (6)	−0.0009 (4)	−0.0008 (5)	0.0004 (4)
C14	0.0268 (7)	0.0143 (5)	0.0191 (6)	0.0023 (5)	−0.0032 (5)	0.0006 (4)
C15	0.0131 (5)	0.0127 (5)	0.0137 (5)	0.0005 (4)	−0.0008 (4)	0.0000 (4)
C16	0.0145 (5)	0.0206 (6)	0.0146 (5)	0.0016 (5)	0.0002 (4)	0.0018 (4)
C17	0.0126 (5)	0.0194 (5)	0.0166 (5)	0.0012 (4)	−0.0004 (4)	0.0010 (4)
C18	0.0136 (5)	0.0140 (5)	0.0156 (5)	0.0011 (4)	−0.0036 (4)	−0.0003 (4)
C19	0.0175 (6)	0.0146 (5)	0.0127 (5)	0.0004 (4)	−0.0011 (4)	−0.0006 (4)
C20	0.0132 (5)	0.0143 (5)	0.0141 (5)	0.0006 (4)	0.0003 (4)	−0.0002 (4)

### Geometric parameters (Å, °)

**Table:**

Br1—C18	1.8988 (13)	C8—C9	1.4052 (19)
Cl1—C11	1.7368 (14)	C9—C10	1.390 (2)
S1—O2	1.4525 (11)	C9—H9A	0.9300
S1—O1	1.4569 (11)	C10—C11	1.382 (2)
S1—O3	1.4572 (10)	C10—H10A	0.9300
S1—C15	1.7820 (13)	C11—C12	1.390 (2)
N1—C1	1.3540 (18)	C12—C13	1.390 (2)
N1—C5	1.3615 (18)	C12—H12A	0.9300
N1—C14	1.4828 (17)	C13—H13A	0.9300
C1—C2	1.373 (2)	C14—H14A	0.9600
C1—H1A	0.9300	C14—H14B	0.9600
C2—C3	1.389 (2)	C14—H14C	0.9600
C2—H2A	0.9300	C15—C20	1.3930 (18)
C3—C4	1.381 (2)	C15—C16	1.3952 (19)
C3—H3A	0.9300	C16—C17	1.3889 (18)
C4—C5	1.4013 (19)	C16—H16A	0.9300
C4—H4A	0.9300	C17—C18	1.3905 (19)
C5—C6	1.4518 (19)	C17—H17A	0.9300
C6—C7	1.3393 (19)	C18—C19	1.3877 (19)
C6—H6A	0.9300	C19—C20	1.3993 (18)
C7—C8	1.4665 (19)	C19—H19A	0.9300
C7—H7A	0.9300	C20—H20A	0.9300
C8—C13	1.4048 (19)		
			
O2—S1—O1	113.69 (7)	C11—C10—C9	118.67 (13)
O2—S1—O3	113.19 (7)	C11—C10—H10A	120.7
O1—S1—O3	112.92 (7)	C9—C10—H10A	120.7
O2—S1—C15	104.47 (6)	C10—C11—C12	121.67 (13)
O1—S1—C15	105.34 (6)	C10—C11—Cl1	118.43 (11)
O3—S1—C15	106.22 (6)	C12—C11—Cl1	119.90 (11)
C1—N1—C5	121.59 (12)	C13—C12—C11	119.18 (13)
C1—N1—C14	117.56 (12)	C13—C12—H12A	120.4
C5—N1—C14	120.84 (11)	C11—C12—H12A	120.4
N1—C1—C2	121.57 (14)	C12—C13—C8	120.85 (13)
N1—C1—H1A	119.2	C12—C13—H13A	119.6
C2—C1—H1A	119.2	C8—C13—H13A	119.6
C1—C2—C3	118.61 (13)	N1—C14—H14A	109.5
C1—C2—H2A	120.7	N1—C14—H14B	109.5
C3—C2—H2A	120.7	H14A—C14—H14B	109.5
C4—C3—C2	119.44 (13)	N1—C14—H14C	109.5
C4—C3—H3A	120.3	H14A—C14—H14C	109.5
C2—C3—H3A	120.3	H14B—C14—H14C	109.5
C3—C4—C5	120.98 (13)	C20—C15—C16	119.85 (12)
C3—C4—H4A	119.5	C20—C15—S1	121.43 (10)
C5—C4—H4A	119.5	C16—C15—S1	118.70 (9)
N1—C5—C4	117.80 (12)	C17—C16—C15	120.59 (12)
N1—C5—C6	118.24 (12)	C17—C16—H16A	119.7
C4—C5—C6	123.95 (13)	C15—C16—H16A	119.7
C7—C6—C5	123.52 (13)	C16—C17—C18	118.74 (13)
C7—C6—H6A	118.2	C16—C17—H17A	120.6
C5—C6—H6A	118.2	C18—C17—H17A	120.6
C6—C7—C8	125.35 (13)	C19—C18—C17	121.86 (12)
C6—C7—H7A	117.3	C19—C18—Br1	119.82 (10)
C8—C7—H7A	117.3	C17—C18—Br1	118.27 (10)
C13—C8—C9	118.10 (12)	C18—C19—C20	118.76 (12)
C13—C8—C7	123.69 (12)	C18—C19—H19A	120.6
C9—C8—C7	118.20 (12)	C20—C19—H19A	120.6
C10—C9—C8	121.50 (13)	C15—C20—C19	120.19 (12)
C10—C9—H9A	119.3	C15—C20—H20A	119.9
C8—C9—H9A	119.3	C19—C20—H20A	119.9
			
C5—N1—C1—C2	−0.7 (2)	C10—C11—C12—C13	0.6 (2)
C14—N1—C1—C2	177.66 (14)	Cl1—C11—C12—C13	−179.20 (11)
N1—C1—C2—C3	−0.2 (2)	C11—C12—C13—C8	0.6 (2)
C1—C2—C3—C4	1.2 (2)	C9—C8—C13—C12	−0.7 (2)
C2—C3—C4—C5	−1.4 (2)	C7—C8—C13—C12	−179.97 (14)
C1—N1—C5—C4	0.5 (2)	O2—S1—C15—C20	132.62 (11)
C14—N1—C5—C4	−177.77 (13)	O1—S1—C15—C20	12.55 (12)
C1—N1—C5—C6	179.13 (13)	O3—S1—C15—C20	−107.47 (11)
C14—N1—C5—C6	0.8 (2)	O2—S1—C15—C16	−45.68 (12)
C3—C4—C5—N1	0.5 (2)	O1—S1—C15—C16	−165.75 (11)
C3—C4—C5—C6	−178.01 (14)	O3—S1—C15—C16	74.23 (12)
N1—C5—C6—C7	177.40 (14)	C20—C15—C16—C17	0.5 (2)
C4—C5—C6—C7	−4.1 (2)	S1—C15—C16—C17	178.87 (11)
C5—C6—C7—C8	178.74 (14)	C15—C16—C17—C18	−0.7 (2)
C6—C7—C8—C13	5.2 (2)	C16—C17—C18—C19	0.1 (2)
C6—C7—C8—C9	−174.16 (15)	C16—C17—C18—Br1	177.48 (10)
C13—C8—C9—C10	−0.4 (2)	C17—C18—C19—C20	0.59 (19)
C7—C8—C9—C10	178.93 (14)	Br1—C18—C19—C20	−176.75 (9)
C8—C9—C10—C11	1.6 (2)	C16—C15—C20—C19	0.17 (19)
C9—C10—C11—C12	−1.7 (2)	S1—C15—C20—C19	−178.11 (10)
C9—C10—C11—Cl1	178.16 (12)	C18—C19—C20—C15	−0.72 (19)

### Hydrogen-bond geometry (Å, °)

**Table:**

*D*—H···*A*	*D*—H	H···*A*	*D*···*A*	*D*—H···*A*
C1—H1A···Br1^i^	0.93	2.89	3.6585 (14)	141
C3—H3A···O3^ii^	0.93	2.53	3.4285 (17)	163
C6—H6A···O2^iii^	0.93	2.55	3.4702 (17)	172
C7—H7A···O1^iv^	0.93	2.51	3.4057 (17)	161
C13—H13A···O2^iii^	0.93	2.49	3.4205 (17)	180
C14—H14A···O1	0.96	2.49	3.3383 (17)	148
C14—H14C···O2^iii^	0.96	2.48	2.9917 (18)	113
C17—H17A···O1^v^	0.93	2.28	3.2110 (17)	175
C20—H20A···O1	0.93	2.55	2.9157 (17)	104
C10—H10A···Cg3^vi^	0.93	2.77	3.6831 (16)	169
C12—H12A···Cg3^iii^	0.93	2.79	3.6402 (16)	153

Symmetry codes: (i) *x*+1, −*y*+3/2, *z*+1/2; (ii) −*x*+2, −*y*+1, −*z*+1; (iii) *x*, −*y*+3/2, *z*−1/2; (iv) −*x*+2, *y*−1/2, −*z*+1/2;  (v) *x*−1, *y*, *z*; (vi) −*x*+1, −*y*+1, −*z*.
